# Assessment of subclinical atherosclerosis and cardiovascular disease in children and adolescents with nephrotic syndrome: a systematic review and meta-analysis

**DOI:** 10.1007/s00467-026-07238-1

**Published:** 2026-03-25

**Authors:** Agni Glava, Anastasia Stoimeni, Nikolaos Gkiourtzis, Maria Kavga, Stella Stabouli, Nikoleta Printza, Despoina Tramma, Kyriaki Papadopoulou Legbelou

**Affiliations:** 1 4th Department of Pediatrics, School of Medicine, Aristotle University of Thessaloniki, “Papageorgiou” General Hospital, Thessaloniki, Greece; 2 3rd Department of Pediatrics, School of Medicine, Faculty of Health Sciences, Aristotle University of Thessaloniki, Hippokration General Hospital, Thessaloniki, Greece; 3 1 st Department of Pediatrics, School of Medicine, Faculty of Health Sciences, Aristotle University of Thessaloniki, Hippokration General Hospital, Thessaloniki, Greece

**Keywords:** Nephrotic syndrome, Children, Subclinical atherosclerosis, Cardiovascular disease, Carotid intima-media thickness, Pulse wave velocity, Left ventricular mass index

## Abstract

**Background:**

Children with nephrotic syndrome (NS) are frequently exposed to chronic inflammation, hypertension, and multiple metabolic abnormalities, such as hyperlipidemia, that may contribute to early development of atherosclerosis and cardiovascular disease. However, the extent of subclinical cardiovascular involvement remains unclear.

**Objectives:**

This systematic review and meta-analysis aimed to investigate the presence of subclinical atherosclerosis and cardiovascular disease in pediatric patients with NS, using carotid intima-media thickness (cIMT), carotid-femoral pulse wave velocity (c-f PWV), and left ventricular mass (LVM) index as surrogate markers.

**Data sources:**

A systematic literature search was conducted in PubMed/MEDLINE, Scopus, and Cochrane Library databases from their inception until December 20, 2024.

**Study eligibility criteria:**

Eligible studies included observational studies that evaluated early markers of atherosclerosis and cardiovascular disease in children with NS.

**Participants and interventions:**

Children and adolescents with NS under the age of 18 years were compared to healthy controls. Surrogate markers included cIMT, PWV, and LVI index. Blood pressure was evaluated as a cardiovascular risk factor.

**Study appraisal and synthesis methods:**

The Newcastle–Ottawa Scale was used to assess study quality. The mean difference with 95% confidence intervals was used for continuous outcomes. Statistical significance was set at *p* < 0.05. Sensitivity and subgroup analyses and meta-regression were conducted for further exploration of the outcomes. Statistical analyses were performed using R software (Version 4.3.2).

**Results:**

Sixteen studies with a total of 1461 participants (668 patients with NS and 793 healthy controls) met inclusion criteria. Children with NS had significantly increased cIMT compared to controls (MD = 0.06 mm; 95% CI: 0.05–0.07; *p* < 0.001), with substantial heterogeneity across studies. No statistically significant differences were observed for c-f PWV and LVM index. Moderate correlations were found between cIMT and disease duration (*r* = 0.47) and number of relapses (*r* = 0.45). A small positive correlation was observed between cIMT and triglycerides, but not with total or LDL cholesterol.

**Limitations:**

This meta-analysis is limited by the observational and predominantly cross-sectional nature of the included studies, substantial heterogeneity across studies, and the highly diverse study population. Furthermore, in many studies, the phenotypes of NS were poorly characterized, or grouped together, and insufficient data were provided for treatment regimens, limiting interpretation of the results.

**Conclusions and implications of key findings:**

Children with NS exhibit increased cIMT, which may be associated with increased future cardiovascular risk. However, prospective longitudinal studies are needed to determine whether these surrogate markers can predict such an association.

**Graphical Abstract:**

**Figure:**
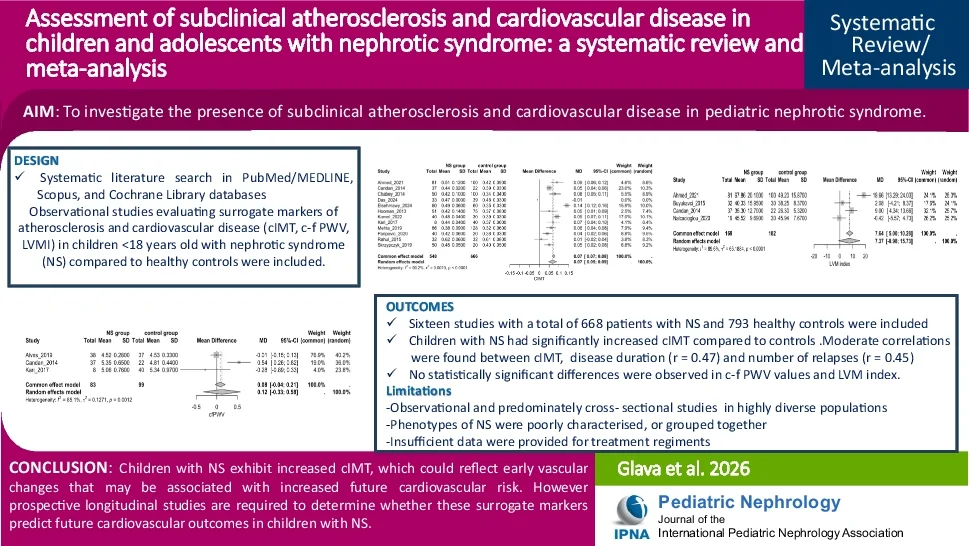
A higher resolution version of the Graphical abstract is available as Supplementary information.

**Supplementary Information:**

The online version contains supplementary material available at https://doi.org/10.1007/s00467-026-07238-1.

## Introduction

Nephrotic syndrome (NS) is one of the most common kidney disorders in children, caused by increased permeability through the damaged basement membrane in the kidney glomeruli [1]. The estimated annual incidence of NS in healthy children is 2–7 new cases per 100,000 children less than 18 years of age [2].

The clinical characteristics of NS include massive proteinuria, which is responsible for hypoalbuminemia, hyperlipidemia, and edema [2]. Administration of corticosteroids is the gold standard of initial treatment in patients with NS [3]. Patients with NS are primarily classified by their response to corticosteroid therapy and the number of relapses [3]. While most patients respond to corticosteroid treatment, some of them may experience relapses, and others are resistant to treatment, requiring immunosuppressive therapy [3, 4].

The relapsing–remitting nature of NS is linked with multiple biochemical alterations that may affect the structure of arterial vessel walls [5]. Clinical and metabolic risk factors that contribute to atherosclerosis in NS include hyperlipidemia, hypertension, nephrotic-range proteinuria, and treatment with corticosteroids or immunosuppressives [4]. Disturbances of lipid metabolism occurring in NS have been reported to persist even during remission periods and may predispose to endothelial dysfunction or structural atherosclerosis [6].

In adults, measurement of carotid intima-media thickness (cIMT) and carotid-femoral pulse wave velocity (c-f PWV) are both reliable surrogate markers of subclinical atherosclerosis of large- and medium-sized vessels, as well as impaired endothelial function and arterial stiffness, which usually precede the onset of cardiovascular disease [7].

We aimed to conduct a systematic review and meta-analysis of the literature, investigating the presence of subclinical atherosclerosis and cardiovascular disease in pediatric patients with NS, using as surrogate markers the cIMT, c-f PWV, and left ventricular mass (LVM) index.

## Methods

### Study registration and search strategy

This systematic review and meta-analysis (SR-MA) followed the Cochrane Handbook for Systematic Reviews of Interventions and adhered to PRISMA guidelines [8]. The protocol was registered in OSF (https://osf.io/ebjnp). We searched PubMed/MEDLINE, Scopus, and Cochrane Library databases from their inception until December 20, 2024 (Supplemental Table 1). A basic search strategy was developed for PubMed/MEDLINE and modified accordingly for other research engines using the terms: (carotid intima-media thickness) OR (cIMT) OR (pulse wave velocity) OR (PWV) OR (left ventricular mass) OR (cardiovascular manifestations) AND (nephrotic syndrome). Reference lists of detected studies were screened for further eligible studies. PROSPERO and OSF databases were also screened to avoid duplicate reviews. Only English studies were included, with no year restrictions.

### Eligibility criteria

Observational studies examining markers of early atherosclerosis and cardiovascular risk in pediatric patients and adolescents under 18 years old with NS who met the eligibility criteria were included in the study. Nephrotic syndrome was defined according to the criteria used in each original study as NS is a heterogeneous clinical disease. Subtypes such as steroid-sensitive (SSNS), steroid-resistant (SRNS), and steroid-dependent (SDNS) nephrotic syndrome were included and documented if data were available. From each study, data on demographic and clinical characteristics, including mean age, sex distribution, disease duration, and treatment exposure (corticosteroids and immunosuppressive agents) were extracted. Additionally, the analysis of vascular outcomes (cIMT, PWV, or LVM), according to treatment type or duration of the disease, was also recorded.

### Study procedure (collection and extraction of data)

Two independent reviewers (A.S. and N.G.) conducted the literature search and data extraction. Records from databases were imported into a reference management tool (rayan.qcri.org), with duplicates removed [9]. Titles and abstracts were screened first, followed by full-text assessment of the remaining articles based on eligibility criteria. Any discrepancies were resolved through discussion with a third author (A.G.) until consensus was reached. Reference lists of included studies, previous systematic reviews, and meta-analyses were manually checked for additional studies. ClinicalTrials.gov, PROSPERO, OSF, and “grey literature” were also searched to identify unpublished, ongoing, or published studies.

Data from the studies included baseline characteristics: first author, year, study name, country, number of participants, groups, sex, and other outcomes. Two authors (A.G. and A.S.) independently extracted this information using a prespecified form. Missing data were obtained by contacting the corresponding authors. Data regarding the selection of control groups were extracted, including the recruitment and matching process.

### Quality assessment of the included studies

The quality of the included studies was evaluated using the modified Newcastle–Ottawa Scale (NOS), assessing population selection, group comparability, and outcome measurement [10]. Studies were classified as low (0–3 points), medium (4–6 points), or high quality (7–9 points). Two independent reviewers (N.G. and A.S.) assessed the risk of bias, resolving disagreements through consensus.

### Outcome measurements

According to the prespecified protocol, the main outcomes included subclinical atherosclerosis and myocardial dysfunction parameters in children with NS, using surrogate markers such as cIMT and c-f PWV for vascular assessment and LVM index as a structural marker of cardiac involvement. Additional outcomes examining the correlation between treatment, duration of the disease, lipid profile, and blood pressure with early markers of atherosclerosis and cardiac disease in children and adolescents with NS were included in our study. BP levels were included in the description of the participants’ baseline cardiovascular profile.

### Statistical analysis and data synthesis

A meta-analysis was conducted using R software (Version 4.3.2) with the “meta” package [11]. Meta-analyses were conducted using a random-effects model (DerSimonian–Laird method) to account for expected heterogeneity among studies. The relative treatment effects were estimated using mean difference (MD) for continuous outcomes with 95% confidence intervals (CI). Forest plots were employed to illustrate the weighted outcomes along with the 95% CI. Publication bias was assessed using funnel plot inspection and Egger’s test when at least ten studies were available for a given outcome. For outcomes with fewer studies, assessment of publication bias was not performed, as such analyses are considered unreliable [12]. Heterogeneity between the studies in pairwise meta-analysis was evaluated using the *I*^2^ test. An *I*^2^ value of less than 40% was considered low, 30–60% as moderate, 50–90% as substantial, and 75–100% as considerable [8]. The *I*^*2*^ thresholds, which were used to describe heterogeneity, are approximate guidelines rather than strict boundaries, as recommended by the Cochrane Handbook. In cases where considerable heterogeneity was observed, a sensitivity analysis was performed [13], followed by further exploration utilizing a Baujat plot [14]. A *p* < 0.05 was deemed statistically significant.

## Results

### Search strategy results and study characteristics

**Fig. 1:**
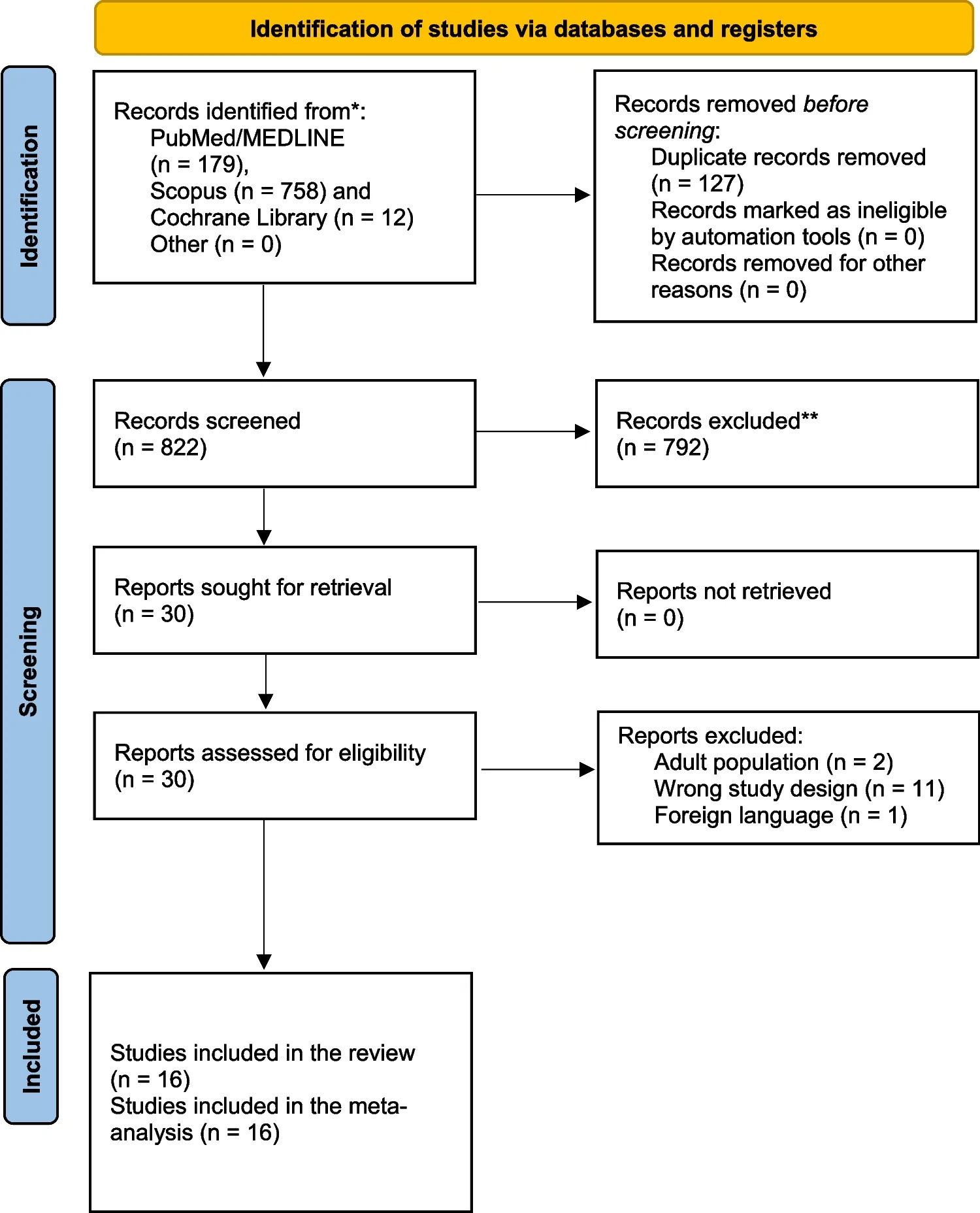
The PRISMA 2020 flow chart. *From:* Page MJ, McKenzie JE, Bossuyt PM, Boutron I, Hoffmann TC, Mulrow CD, et al. The PRISMA 2020 statement: an updated guideline for reporting systematic reviews. BMJ 2021;372:n71. 10.1136/bmj.n71. For more information, visit: http://www.prisma-statement.org/

In total, we identified 949 records from our initial search. After duplicate removal and title and abstract screening, 30 studies remained for full-text assessment, with 16 studies being eligible for our systematic review (Fig. 1). Excluded studies are summarized in Supplemental Table 2. In total, sixteen studies with 1461 participants (668 pediatric patients with NS and 793 controls) met the inclusion criteria [15–30] (Table 1). While our search strategy encompassed all available years in PubMed, Scopus, and Web of Science, no eligible studies prior to 2013 were identified. This ensures that the analysis focuses on studies reflecting current treatment approaches and diagnostic practices.

**Table 1:** Baseline characteristics of the included studies

*Study ID*	*Registration number*	*Country*	*Type of study*	*Mean age (SD)*	*Male (%)*	*Patients included in the study*	*Study groups*	*Disease duration*	*Mean BMI (kg/m*^*2*^*)*	*Response to corticosteroids (yes/no)*	*CIMT (mm)*	*Mean LVM (gm)*	*Mean LVM index (g/m2)*	*c-f PWV (m/s)*	*Number of relapses (mean, SD)*	*Medication*
* Ahmed 2021*	FMBSUREC/05032019 (FWA00015574)	Egypt	Case–control	**Cases**: 8.8 years (3.44) **Controls**: 7.65 years (3.66)	**Cases**: 66.7 **Controls**:70	**Cases**: 81 **Controls**: 100	**NS: **SSNS SDNS SRNS **Controls**	3.6 years (2.6)	**Cases**: 17.55 (3.18) **Controls**: 16.26 (1.46)	Both	**Cases**:0.51 (0.12) **Controls**: 0.42 (0.09)	**Cases**: 70.38 (24.59) **Controls**: 44.85 (11.96)	**Cases**: 67.86 (20.1) **Controls**: 49.2 (15.87)	N/A	2.1 ± 1.9	42% immunosuppression: 19.7% cyclophosphamide, 24.7% CNI, 6.2% MMF, 6% combined (CNI + MMF)
* Alves 2019*	FCMMG CAAE 47152815.7.00005134 and CAAE 47152815.7.3001.5149	Brazil	Cross-sectional	**Cases**: 12.1 years (3.65) **Controls**: 13.3 years (2.80)	**Cases**: 65.7 **Controls**: 56.75	**Cases**: 38 **Controls**: 37	**Primary NS** **Controls**	N/A	N/A	Both	N/A	N/A	N/A	**Cases**: 4.52 (0.28) **Controls**: 4.53 (0.33)	N/A	100% corticosteroid, 42.11% cyclosporine
*Büyükavcı 2015*	28.05.2009/41	Turkey	Cross-sectional	**Cases**: 7.78 years (5.17) **Controls**: 9.47 years (4.17)	**Cases**: 75 **Controls**: 76.67	**Cases**: 32 **controls**: 30	**NS** (hypovolemic and nonhypovolemic) **Controls**	N/A	N/A	Yes	N/A	N/A	**Cases**: 40.33 (15.95) **Controls**: 38.25 (8.37)	N/A	N/A	No steroids/immunosuppressive drugs in previous 3 months
* Candan 2014*	N/A	Turkey	Cross-sectional	**Cases**: 13.9 years (3.2) **Controls**: 13.3 years (2.5)	**Cases**: 48.64 **Controls**: 45.45	**Cases**: 37 **Controls**: 22	**SRNS** **Controls**	94.5 months (59.4)	**Cases**: 21.1 (4.60) **Controls**: 19.7 (2.94)	No	**Cases**: 0.44 (0.02) **Controls**: 0.39 (0.03)	**Cases**: 105 (42.4) **Controls**: 76.2 (21.6)	**Cases**: 35.3 (12.7) **Controls**: 26.3 (5.32)	**Cases**: 5.35 (0.65) **Controls**: 4.81 (0.44)	N/A	45.9% low-dose corticosteroids, 13% pulse corticosteroids, 40% cyclosporine, 70% CNI, 32% CNI + ARBs
*Chabey 2014*	N/A	India	Cross-sectional	N/A	**Cases**:60 **Controls**: 60	**Cases**:50 **Controls**: 100	**NS** **Controls**	N/A	**Cases**: 17.71 (1.7) **Controls**: 16.49 (1.64)	Both	**Cases**: 0.42 (0.1) **Controls**: 0.34 (0.04)	N/A	N/A	N/A	N/A	N/A
* Das 2024*	N/A	India	Cross-sectional	**Cases**: 7.21 years (2.83) **Controls**: 9.92 years (2.76)	**Cases**: 69 **Controls**:49	**Cases**:33 **Controls**:39	**NS** **Controls**	34.7 months (24)	**Cases**: 16.38 (2.58) **Controls**: 15.77 (2.63)	Both	**Cases**: 0.47 (0.0) **Controls** 0.48 (0.03)	N/A	N/A	N/A	4 ± 2.27 (SD)	15% cyclosporine, 33% MMF, 3% tacrolimus, 9% levamisole
* Elsehmawy 2024*	202,107,919	Egypt	Case–control	**Cases**: 9.6 years (3.6) **Controls**: 9.7 years (3.7)	**Cases**: 65 **Controls**: 71.67	**Cases**: 60 **Controls**: 60	**NS** **Controls**	2.9 years (2.6)	**Cases**:21.3 (5.6) **Controls**: 20.1 (3.8)	Both	**Cases**: 0.49 (0.06) **Controls**: 0.35 (0.03)	N/A	N/A	N/A	N/A	N/A
* Hooman 2013*	N/A	Iran	Cross-sectional	**Cases**: 7.8 years (4) **Controls**: 7.9 years (3)	**Cases**: 67 **Controls**: 53	**Cases**: 51 **Controls**:75	**NS** **Controls**	4.7 years (7.6)	**Cases**:18.7 (5.5) **Controls**: 16.2 (3.5)	Both	**Cases**: 0.42 (0.14) **Controls**: 0.37 (0.08)	N/A	N/A	N/A	N/A	100% corticosteroids, 23.5% cyclophosphamide, 37% cyclosporine, 14% MMF, 25.5% levamisole, 31% ACEI, 16% ARB, 47% lovastatin Levostatin:47%
* Kamel 2022*	N/A	Egypt	Case–control	**Cases**: 8.5 years (3.5) **Controls**: 7.03 years (3.3)	**Cases**: 72.5 **Controls**: 60	**Cases**: 40 **Controls**: 30	**NS:** SSNS SDNS SRNS **Controls**	3.2 years (2.5)	**Cases**:17.9 (2.8) **Controls**: 15.8 (2.2)	Both	**Cases**: 0.48 (0.04) **Controls**: 0.39 (0.03)	N/A	N/A	N/A	SSNS: 3 ± 1 SDNS: 4 ± 2 SRNS: 1 ± 1	30% CNI, 15% MMF, 2.5% cyclophosphamide, 12.5% combined (MMF + CNI + CYP)
* Kari 2017*	N/A	United Kingdom	Case–control	**Cases**: 10.8 years (4.2) **Controls**: 13.2 years (4.8)	**Cases**: 37.5 **Controls**: 51.28	**Cases**: 8 **Controls**:39	**SRNS** **Controls**	40.9 months (20.7)	**Cases**: 21.8 (4.0) **Controls**: N/A	No	**Cases**: 0.44 (0.04) **Controls**: 0.37 (0.59)	N/A	N/A	**Cases**: 5.06 (0.76) **Controls**: 5.34 (0.97)	**N/A**	**N/A**
	N/A	India	Case–control	**Cases**: 6.71 years (3.4) **Controls**: 7.89 years (3.95)	**Cases**: 60.6 **Controls**: 59.37	**Cases**: 66 **Controls**: 128	**NS** **Controls**	2.39 years (1.44)	N/A	Both	**Cases**: 0.38 (0.09) **Controls**: 0.32 (0.06)	N/A	N/A	N/A	3.46 ± 1.34	N/A
*Nalcalioglu 2020*	N/A	Turkey	Case–control	**Cases**: 5.08 years (2.41) **Controls**: 6.68 years (3.04)	**Cases**: 57.89 **Controls**: 40	**Cases**: 19 **Controls**: 30	**NS** **Controls**	N/A	**Cases**: (debut/relapses) 18.84 (2.37) (remission) 18.59 (2.14) **Controls**: 17.31 (2.46)	Yes	N/A	N/A	**Cases**: (debut/relapses) 43.28 (9.94) (remission) 45.52 (9.69) **Controls**: 45.94 (7.67)	N/A	N/A	Corticosteroids
*Papirovic 2020*	N/A	Serbia	Cross-sectional	**Cases**: 11.7 years (4.7) **Controls**: 12.7 years (3.7)	**Cases**: 72.5 **Controls**: 55	**Cases**:40 **Controls**: 20	**NS:** SDNS SRNS **Controls**	6.4 years (4.5)	**Cases**: 21.0 (4.4) **Controls**: 21.2 (4.5)	Both	**Cases**: 0.42 (0.06) **Controls**: 0.38 (0.03)	N/A	N/A	N/A	> 5 relapses: 30% patients, 5–10 relapses: 27.5% > 10 relapses 37.5%	15% prednisone, 12.5% prednisone + cyclosporine A, 5% cyclosporine A
* Rahul 2015*	SEC/2011/4/90	India	Cross-sectional Case–control	**Cases**: 8.9 years (2.8) **Controls**: 9.0 years (2.8)	**Case**s:59.4 **Controls**: 50	**Cases**: 32 **Controls**: 32	**NS**: IFRNS FRNS SDNS SRNS **Controls**	5.29 years (2.09)	**Cases**: 16.6 (2.8) **Controls**: 14.7 (1.9)	Both	**Cases**: 0.62 (0.06) **Controls**: 0.61 (0.08)	N/A	N/A	N/A	13.4 ± 5.9	25% prednisolone, 59.4% levamisole + prednisolone, 43.8% cyclophosphamide + prednisolone, 25% cyclosporine + prednisolone
* Skrzypczyk 2019*	N/A	Poland	Cross-sectional	**Cases**: 9.47 years (4.44) **Controls**: 9.46 years (2.29)	**Cases**: 64 **Controls**: 65	**Cases**: 50 **Controls**: 20	**NS** **Controls**	6.41 years (4.83)	N/A	Both	**Cases**: 0.45 (0.05) **Controls**: 0.40 (0.05)	N/A	N/A	N/A	7.14 ± 5.31	56% prednisone, 24% cyclosporin A, 12% MMF
* Youssef 2018*	N/A	Egypt	Case–control	**Cases**: 6.13 years (0.76) **Controls**: 6.03 years (0.83)	**Cases**: 61.29 **Controls**: 61.29	**Cases**: 31 **Controls**: 31	**NS** **Controls**	N/A	**Cases**: 19.28 (2.88) **Controls**: 16.14 (1.31)	Both	N/A	N/A	N/A	N/A	N/A	N/A

*FRNS*, frequently relapsing nephrotic syndrome; *IFRNS*, infrequently relapsing nephrotic syndrome; *N/A*, not applicable; *NS*, nephrotic syndrome; *SDNS*, steroid-dependent nephrotic syndrome; *SRNS*, steroid-resistant nephrotic syndrome; SSNS, steroid-sensitive nephrotic syndrome; *CIM*, carotid intima-media thickness; *LVM*, left ventricular mass; *c-f PWV*, carotid-femoral pulse wave velocity; *BMI*, body mass index; *MMF*, mycophenolate mofetil; *CNI*, calcineurin inhibitors; *ARB*, angiotensin receptor blockers; *ACEI*, ACE inhibitors

In the included studies, children with NS and control groups were matched for age and sex. However, three exceptions were noted: in the study by Das et al*.* [20], they were matched for sex but not for age, while in the studies by Kari et al*.* [24] and Rahul et al*.* [29] the groups were matched for age but not for sex (Supplemental Table 3).

### Quality assessment of the included studies

The quality of the included studies was assessed using the modified NOS, which evaluates three domains: selection of participants, comparability of study groups, and ascertainment of exposure or outcome. Twelve of the 16 studies included in our study were evaluated to be of “high quality” [15–22, 26, 28–30] and four out of 16 were evaluated as “medium quality” [23–25, 27]. A summary of the quality assessment (NOS scoring) is described in Table 2.

**Table 2:** Quality assessment of the included studies with Newcastle–Ottawa Scale

*First author & year*	*Is the case definition adequate?*	*Representativeness of the cases*	*Selection of controls*	*Definition of controls*	*Comparability of cases and controls based on the design or analysis*	*Ascertainment of exposure*	*Same method of ascertainment for cases and controls*	*Nonresponse rate*	*Total score*
* Ahmed 2021*	*	*	*	*	*	*	*		7/9
* Alves 2019*	*	*	*	*	**	*	*		8/9
*Büyükavcı 2015*	*	*	*	*	*	*	*		7/9
* Candan 2014*	*	*	*	*	**	*	*		8/9
*Chabey 2014*	*	*	*	*	*	*	*		7/9
* Das 2024*	*	*	*	*	*	*	*		7/9
* Elsehmawy 2024*	*	*	*	*	*	*	*		7/9
* Hooman 2013*	*	*	*	*	*	*	*		7/9
* Kamel 2022*	*	*	*	*		*	*		6/9
* Kari 2017*	*	*	*			*	*		5/9
* Mehta 2019*	*	*	*		*	*	*		6/9
* Nalcacioglu 2020*	*	*	*	*	*	*	*		7/9
*Paripovic 2020*	*	*	*	*	*	*	*		7/9
* Rahul 2015*	*	*	*	*		*	*		7/9
* Skrzypczyk 2019*	*	*	*	*	*	*	*		7/9
* Youssef 2018*	*	*	*			*	*		5/9

### Analysis of primary outcomes

#### Carotid intima-media thickness (cIMT)

**Fig. 2:**
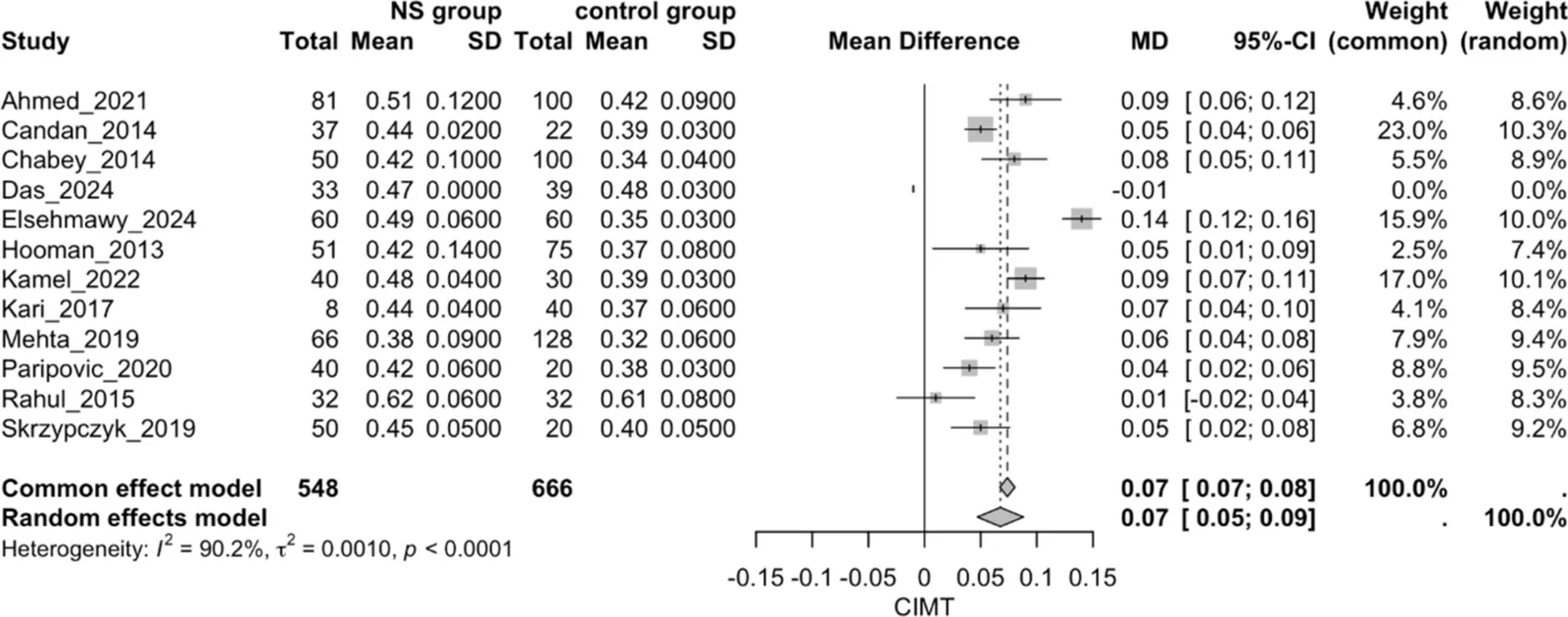
Forest plot which evaluates the difference in cIMT between patients and controls. cIMT: carotid intima-media thickness

**Fig. 3:**
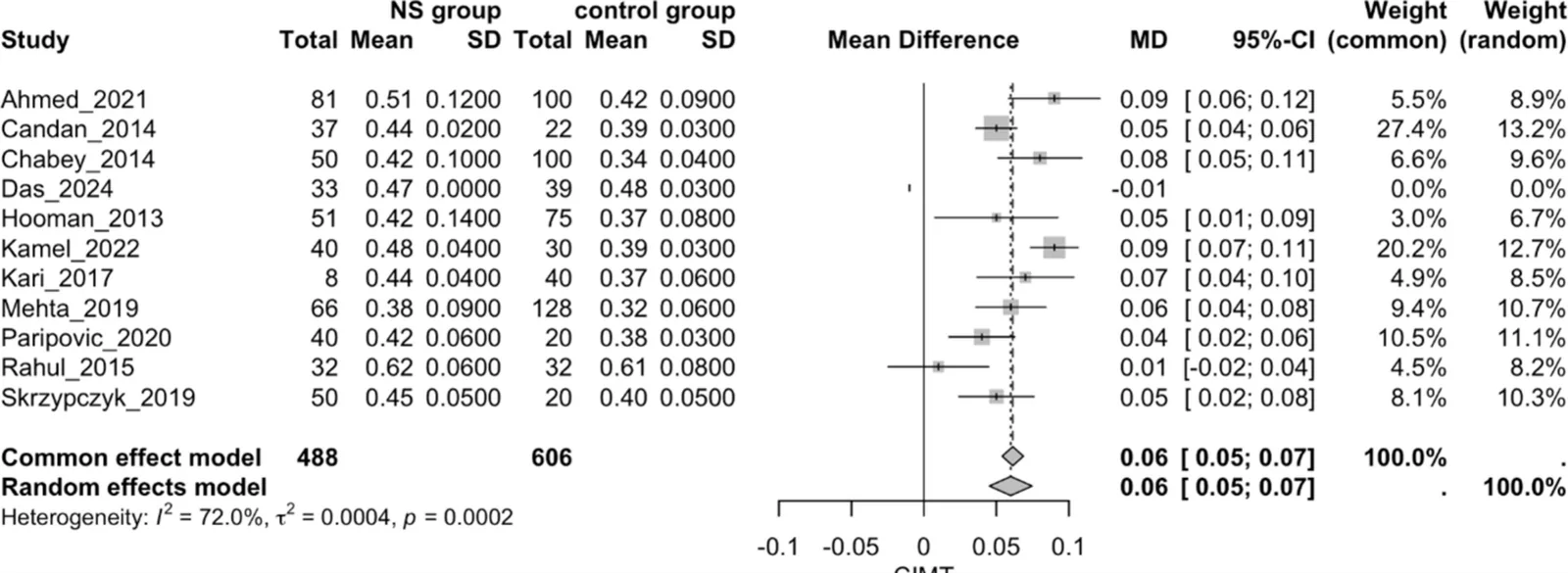
Forest plot assessing the difference in cIMT between patients and controls after sensitivity analysis. cIMT: carotid intima-media thickness

**Fig. 4:**
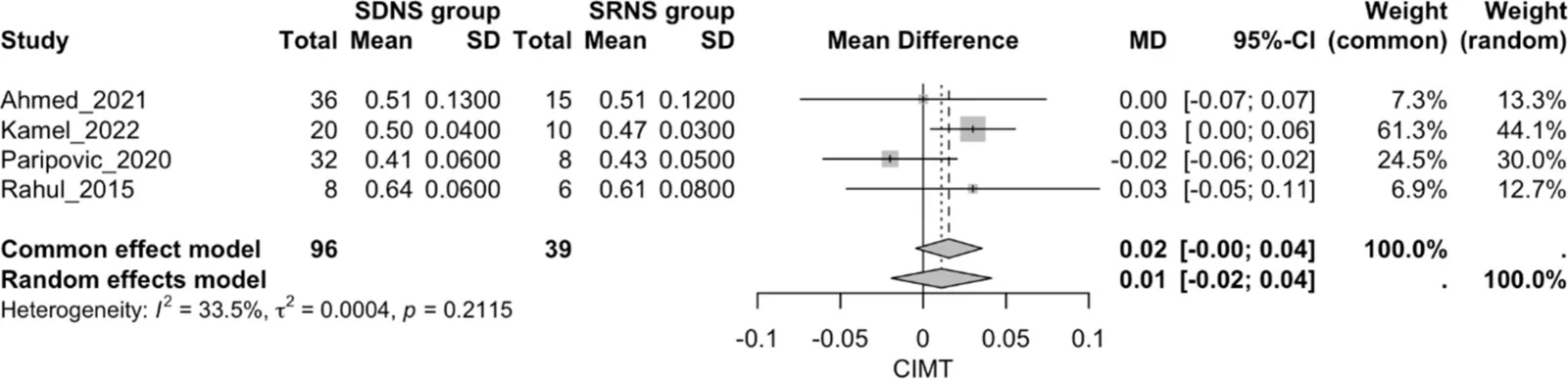
Forest plot assessing the difference in cIMT between patients with steroid-dependent and steroid-resistant nephrotic syndrome. cIMT: carotid intima-media thickness

Overall, 12 studies assessed cIMT, including patients with NS, regardless of their response to steroids [15, 18–21, 23–25, 27–30]. The patient group exhibited statistically increased cIMT compared to the control group (MD = 0.07, 95% CI [0.05, 0.09], *p* < 0.0001), but with substantial heterogeneity (*I*^2^ = 90.2%) (Fig. 2). Following a sensitivity analysis, the study of Elsehmawy et al*.* was omitted as it was identified as the primary source of heterogeneity (Supplemental Fig. 1). This finding was further supported by the Baujat plot analysis (Supplemental Fig. 2). After this adjustment, the patient group still showed significantly increased cIMT compared to the control group (MD = 0.06, 95% CI [0.05, 0.07], *p* < 0.001), with reduced heterogeneity (*I*^2^ = 72%) (Fig. 3). No publication bias was identified through Egger’s test (*p* = 0.22) (Supplemental Fig. 3). Furthermore, a subgroup analysis comparing steroid-dependent (SDNS) and steroid-resistant NS (SRNS) was conducted, including four studies [15, 23, 29, 30]. The findings revealed no statistically significant difference in cIMT between the SDNS and SRNS groups (MD = 0.01, 95% CI [−0.02, 0.04], *p* = 0.48, *I*^2^ = 33.5%) (Fig. 4).

Mixed-effects meta-regressions were conducted to explore whether mean age or disease duration influenced the outcome of the studies. Both meta-regression models were fitted using a mixed-effects model approach with the restricted maximum likelihood (REML) estimator. In the first model, which included mean age as a moderator, there was substantial residual heterogeneity (*I*^2^ = 89.42%), and the test for residual heterogeneity remained highly significant (*p* < 0.0001). The moderator mean age did not significantly explain the between-study variability (*p* = 0.82), and the amount of heterogeneity accounted for (*R*^2^) was 0%. The regression coefficient for mean age (estimate =  − 0.0014, 95% CI: − 0.013 to 0.01) was not statistically significant, suggesting that the effect size did not vary systematically with the mean age of participants (Supplemental Fig. 4). 

In contrast, the second model, which included disease duration as a moderator, showed improved model fit with a moderate reduction in heterogeneity (*I*^2^ = 83.91%; *R*^2^ = 30.82%), indicating that approximately one-third of the between-study variance was explained by disease duration. The test for residual heterogeneity remained significant (*p* < 0.0001), implying that additional sources of heterogeneity were still present. The test of moderators was statistically significant (*p* = 0.038), and the regression coefficient for disease duration was also significantly negative (estimate =  − 0.0114, 95% CI: − 0.022 to − 0.0006), indicating that longer disease duration was associated with smaller effect sizes (Supplemental Fig. 5).

### Carotid-femoral pulse wave velocity (c-f PWV)

**Fig. 5:**
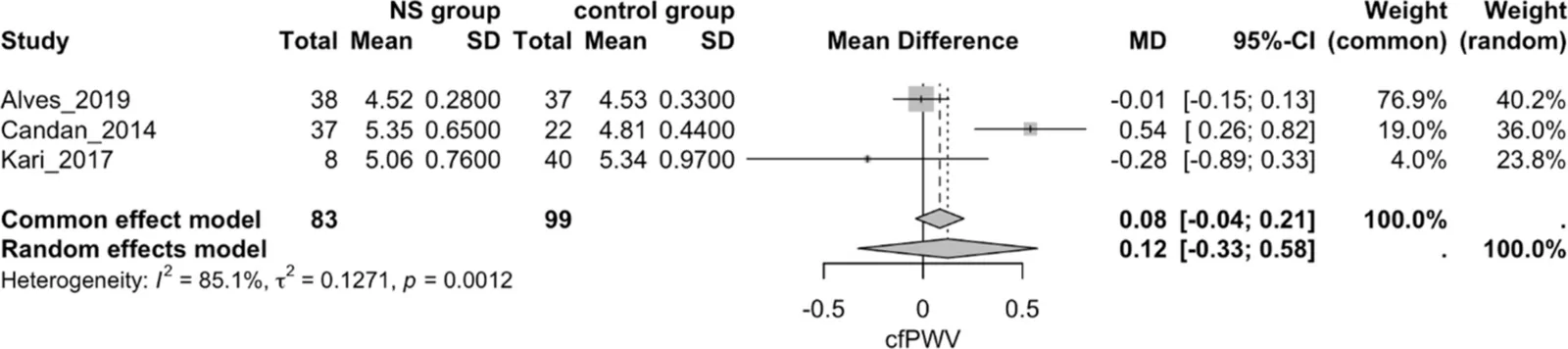
Forest plot assessing the difference in PWV between patients and controls. PWV: pulse wave velocity

Three studies evaluated c-f PWV [16, 24,18]. The patient group exhibited higher PWV values, compared to the control group (MD = 0.12, 95% CI [−0.33, 0.58], *p* = 0.5, *I*^2^ = 85.1%), though the difference was not statistically significant (Fig. 5).

### Left ventricular mass (LVM) index

**Fig. 6:**
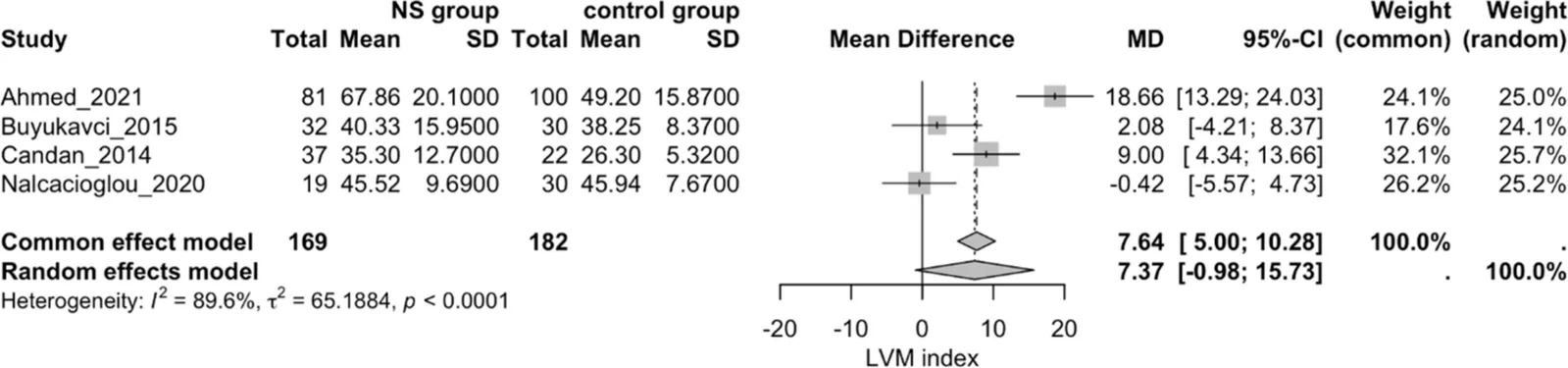
Forest plot assessing the difference in LVM index between patients and controls. LVM: left ventricular mass

Four studies evaluated the LVM index [15, 17, 18, 26]. The patient group exhibited higher LVM index values compared to the control group (MD = 7.37, 95% CI [−0.98, 15.73], *p* = 0.08, *I*^2^ = 89.6%), though the difference was not statistically significant (Fig. 6).

### Analysis of BP as a baseline characteristic

#### Systolic blood pressure (SBP)

A total of eight studies assessed SBP values [15, 16, 19–22, 26, 27]. Only two studies employed ABPM [18, 30]. BP levels in the remaining studies are based on office measurements. The findings indicated that the patient group had significantly higher SBP values, compared with controls (MD = 3.02, 95% CI [0.73, 5.31], *p* = 0.01, *I*^2^ = 62.8%) (Supplemental Fig. 6). After omitting the study by Elsehmawy et al*.* (Supplemental Fig. 7), the heterogeneity was restricted. However, the SBP values of patients with NS remained significantly higher, compared with controls (MD = 3.72, 95% CI [1.62, 5.83], *p* = 0.005, *I*^2^ = 46.9%) (Supplemental Fig. 8).

### Diastolic blood pressure (DBP)

Eight studies evaluated DBP values [15, 16, 19–22, 26, 27]. The patient group did not demonstrate significantly higher DBP values compared with controls (MD = 2.32, 95% CI [−1.18, 5.83], *p* = 0.19, *I*^2^ = 86.1%) (Supplemental Fig. 9). A sensitivity analysis was conducted by excluding the study of Yousef et al*.* (Supplemental Fig. 10)*.* After this adjustment, the patient group exhibited significantly higher DBP values compared to the control group (MD = 3.60, 95% CI [0.98, 6.21], *p* = 0.007, *I*^2^ = 67.9%) (Supplemental Fig. 11).

### Analysis of correlations—secondary outcomes

#### Correlation of cIMT with duration of disease in patients with NS

Seven studies analyzed the correlation of cIMT and the duration of disease [15, 19, 21, 23, 25, 28, 30]. The correlation coefficients varied from −0.05 to 0.83. The estimated Fisher *r*-to-*z*-transformed correlation coefficient, depending on the random-effects model, was *r* = 0.47 (95% CI [0.22–0.66], *p* = 0.0004, *I*^2^ = 86.5%), suggesting a moderate positive relationship between the two variables (Supplemental Fig. 12).

### Correlation of cIMT with relapses in patients with NS

Three studies analyzed the correlation of cIMT and relapses in the patient group [23, 25, 28]. The correlation coefficients varied from 0.38 to 0.51. The estimated Fisher *r*-to-*z*-transformed correlation coefficient, depending on the random-effects model, was r = 0.45 (95% CI [0.31–0.57], *p* < 0.0001, *I*^2^ = 0%), suggesting a moderate positive relationship between the two variables (Supplemental Fig. 13).

### Correlation of cIMT with lipid profile

Five studies analyzed the correlation between cIMT and triglycerides [15, 19, 21, 23, 25]. The correlation coefficients varied from 0.06 to 0.48. The estimated Fisher *r*-to-*z*-transformed correlation coefficient, depending on the random-effects model, was *r* = 0.24 (95% CI [0.08–0.38], *p* = 0.003, *I*^2^ = 78.2%), suggesting a small positive correlation (Supplemental Fig. 14).

No significant correlation was detected between cIMT and total cholesterol [15, 19, 21, 23, 25] or LDL-cholesterol [15, 19, 25] (Supplemental Fig. 15 and 16).

### Correlation of cIMT with baseline characteristics

No significant correlation was detected between cIMT and SBP, DBP, or BMI (Supplemental Fig. 17, 18, and 19).

## Discussion

Evaluation of cIMT is a reliable and widely used method in adults to determine endothelial dysfunction [31]. However, its significance in the pediatric population is still controversial, with an increasing body of research involving children with risk factors for vascular damage [32]. Endothelial dysfunction is the initial, potentially reversible stage of atherosclerosis, predicting cardiovascular disease [33]. It is characterized by fibrosis of the intima and calcification of the media layers of carotid arteries [32]. PWV is used for the evaluation of functional vascular changes, such as arterial stiffness [34, 35]. Arterial stiffness of the large arteries, measured by the c-f PWV, is considered one of the main factors affecting cardiovascular risk in the hypertensive population and has been included in hypertension screening guidelines of the European Society of Cardiology and European Society of Hypertension (ESC/ESH) [36].

Recent studies suggest that increased cIMT in children with obesity, hypertension, and dyslipidemia correlate with early vascular remodeling and may predict higher cardiovascular risk in adulthood [37, 38]. Elevated c-f PWV values in children with metabolic or kidney disease have also been associated with subclinical cardiovascular damage and an unfavorable cardiometabolic profile [37, 39]. Furthermore, increased LVMI values have been observed in children with hypertension, as well as in children with chronic kidney disease (CKD) and they are associated with early target organ damage and a greater likelihood of cardiovascular complications later in life [40, 41].

Pediatric patients with NS may be at greater risk for atherosclerosis and cardiovascular disease, due to recurrent exposure to well-known atherogenic factors such as lipid abnormalities, high blood pressure, coagulation abnormalities, and inflammation [6, 42, 43]. Furthermore, children with SRNS may progress towards CKD, which is known to increase cardiovascular mortality by 500–1000-fold in young adults, compared to the general population [44].

To our knowledge, this is the first systematic review and meta-analysis investigating the presence of subclinical atherosclerosis and cardiovascular disease in pediatric patients with NS. The results of our study showed that children with NS exhibited statistically significantly higher cIMT values compared to the control group. In addition, the patient group exhibited higher PWV values and LVM index compared to the control group, but these differences did not reach statistical significance. Our results also demonstrate that there is a statistically significant correlation between the duration of the disease, the number of relapses, triglyceride levels, and cIMT. However, there was no significant correlation between total cholesterol or LDL-cholesterol levels and cIMT. Therefore, our findings show that children with NS exhibit increased cIMT, which could reflect early vascular changes that may associate with increased future cardiovascular risk. However, further evidence linking cIMT to future cardiovascular outcomes in this population needs to be explored by future prospective longitudinal studies. This hypothesis is further supported by the study conducted by Bharati et al*.* involving 101 patients with childhood-onset NS, aged over 15 years, who were either in long-term remission or continued to have recurrence of the disease with long-term complications. They reported an increased cIMT in 50.5% of all patients, of whom 35.3% were in the long-term remission group and 64.7% in the group without long-term remission, suggesting that cardiovascular disease and atherosclerosis are long-term complications of NS [45].

Our study exhibits several limitations. Substantial heterogeneity was present in our analysis and could be related to the sample size, differences in age, sensitivity to treatment with corticosteroids, and treatment options (duration of treatment, response to corticosteroids, treatment with immunosuppressants, or immunomodulators). In our meta-analysis, differences in participants’ mean age across studies did not contribute meaningfully to the observed heterogeneity, whereas disease duration emerged as a significant moderator. More specifically, the negative association between disease duration and effect size indicates that the observed biomarker differences tended to diminish with longer disease duration, potentially reflecting adaptive physiological changes or treatment effects over time. Moreover, most control groups were carefully selected to be healthy and comparable to the patient groups, but the heterogeneity in control selection and matching criteria across studies may introduce selection bias, potentially leading to over- or underestimation of the group differences.

In many studies, the NS phenotypes were poorly characterized or grouped together. Another limitation is both the lack of detailed information on treatment subgroups within the included studies and the lack of data regarding timing of measuring lipid levels. Specifically, data were insufficient to evaluate cIMT and other cardiovascular outcomes in children treated with steroids alone, compared with those receiving calcineurin inhibitors, cyclophosphamide, or rituximab. Another important limitation is that most studies do not report vascular outcomes separately for NS subtypes, conferring high heterogeneity between studies and not allowing further stratified analysis. Interpretation of our subgroup analyses by disease duration and number of relapses is also limited, as these measures do not fully reflect the disease activity or the impact on cardiovascular risk. Moreover, we could not assess the relationship between the surrogate markers and risk factors we used, such as proteinuria, blood pressure and kidney function during the relapses, due to a lack of efficient data. Last but not least, the number of eligible studies was relatively limited, and all of them were observational cross-sectional, without long-term follow-up. This may limit the ability to infer causality between nephrotic syndrome and early markers of atherosclerosis. Future longitudinal studies are needed to confirm and extend these findings. Finally, our results cannot be compared with those of adults with NS, as no sufficient bibliographic data could be found.

Nevertheless, our study includes a large pool of participants (*n* = 1461), and the quality of most of the studies included was high. So, despite the study’s limitations, our findings suggest that children with NS should be in close monitoring for early atherosclerosis and cardiovascular disease, especially those with an extended duration of disease and those with multiple relapses. In clinical practice, this could include periodic assessment of blood pressure measurement, serum lipids, and renal function tests as part of routine follow-up, as well as noninvasive vascular evaluation (such as cIMT measurement) in high-risk patients but evidence is still needed before suggesting routine evaluation of these markers.

The above statement is supported by Kniazewska et al*.,* who highlighted the correlation between cIMT and the number of recurrences, suggesting that patients treated for idiopathic NS should undergo regular laboratory tests for atherosclerosis risk factors, especially patients with numerous relapses [6].

Therefore, despite the significant limitations, this is a meta-analysis focusing on subclinical atherosclerosis in children with NS based on studies published after 2013, an underexplored and highly relevant topic. This is a great advantage as it highlights the necessity of comparing data on a hot topic of CVD in NS.

## Conclusion

This study represents the first systematic review and meta-analysis examining the presence of subclinical atherosclerosis and cardiovascular disease in children and adolescents with NS. Our findings show that children with NS exhibit increased cIMT, which could reflect early vascular changes and may be associated with increased future cardiovascular risk. However, further prospective studies are needed to confirm its predictive value for future cardiovascular events. Despite the presence of limitations and limited data for certain outcomes, the inclusion of 1461 participants (668 pediatric patients with NS and 793 controls) increases the reliability of our results. Further well-designed clinical studies investigating novel laboratory and imaging markers of atherosclerosis would be crucial for assessing the cardiovascular risk in these patients.

## Supplementary Information

Below is the link to the electronic supplementary material.

ESM 1 Graphical abstract (335 KB)

ESM 2 (PNG 213 KB)

ESM 3 (PNG 75.0 KB)

ESM 4 (PNG 69.4 KB)

ESM 5 (PNG 75.0 KB)

ESM 6 (PNG 79.5 KB)

ESM 7 (PNG 216 KB)

ESM 8 (PNG 177 KB)

ESM 9 (PNG 199 KB)

ESM 10 (PNG 87.8 KB)

ESM 11 (PNG 143 KB)

ESM 12 (PNG 181 KB)

ESM 13 (PNG 141 KB)

ESM 14 (PNG 158 KB)

ESM 15 (PNG 137 KB)

ESM 16 (PNG 156 KB)

ESM 17 (PNG 2148 KB)

ESM 18 (PNG 148 KB)

ESM 19 (PNG 165 KB)

ESM 20 (DOCX 14.1 K)

ESM 21 (DOCX 19.5 KB)

ESM 22 (DOCX 18.5 KB)

ESM 23 (PNG 234 KB)

## Data Availability

The datasets generated during and/or analyzed during the current study are available from the corresponding author on reasonable request.
