# Supplementary material for: Assessment of subclinical atherosclerosis and cardiovascular disease in children and adolescents with nephrotic syndrome: a systematic review and meta-analysis
Source: Pediatr Nephrol. 2026 Mar 25;41(11):3853–66. doi: 10.1007/s00467-026-07238-1 (PMC13612619; doi:10.1007/s00467-026-07238-1)
Supplement: Supplementary file 1 [file 467_2026_7238_MOESM20_ESM.docx]

**Number of records identified through searching electronic databases**

**PubMed/MEDLINE** (20/12/2024)

(((((((carotid intima media thickness) OR (CIMT)) OR (left ventricular mass)) OR (LVM)) OR (cardiac manifestations)) OR (pulse wave velocity)) OR (PWV)) AND (nephrotic syndrome)

**Results**: 179

**Scopus** (20/12/2024)

( "carotid intima thickness" ) OR ( "CMT" ) OR ( "left ventricular mass" ) OR ( "LVM" ) OR ( "cardiac manifestations" ) OR ( "pulse wave velocity" ) OR ( "PWV" ) AND ( "nephrotic syndrome" )

**Results:** 758

**Cochrane Library** (20/12/2024)

"carotid intima media thickness" in Title Abstract Keyword OR left ventricular mass in Title Abstract Keyword OR cardiac manifestations in Title Abstract Keyword OR pulse wave velocity in Title Abstract Keyword AND nephrotic syndrome in Title Abstract Keyword - (Word variations have been searched)

**Results:** 12

127 duplicates
