# Supplementary material for: Assessment of subclinical atherosclerosis and cardiovascular disease in children and adolescents with nephrotic syndrome: a systematic review and meta-analysis
Source: Pediatr Nephrol. 2026 Mar 25;41(11):3853–66. doi: 10.1007/s00467-026-07238-1 (PMC13612619; doi:10.1007/s00467-026-07238-1)
Supplement: Supplementary file 2 [file 467_2026_7238_MOESM21_ESM.docx]

| **Study ID** | | **Title** | **Reasons for exclusion** |
| --- | --- | --- | --- |
| 1 | Adenoyin 2004 | Cardiac disease in children with primary glomerular disorders—role of focal segmental glomerulosclerosis | Wrong study design |
| 2 | Akinyosoye 2022 | Prevalence and determinants of peripheral arterial disease in children with nephrotic syndrome | Wrong study design |
| 3 | Aksenova 2016 | Risk factors for myocardial remodeling in children with steroid-resistant nephrotic syndrome | Wrong study design |
| 4 | Alegria-Torres 2014 | Cardiovascular risk factors in children with primary nephrotic syndrome | Foreign Language |
| 5 | Bharati 2023 | Long-term complications in patients with childhood-onset nephrotic syndrome | Adult population |
| 6 | Frishberg 2006 | The Heart of Children with Steroid-Resistant NephroticSyndrome: Is It All Podocin? | Wrong study design |
| 7 | Hafez 2021 | Evaluation of Biochemical Bone Markers in Children with Nephrotic Syndrome with Correlation to Its Severity | Wrong study design |
| 8 | Hari 2018 | Effect of atorvastatin on dyslipidemia and carotid intima-media thickness in children with refractory nephrotic syndrome: a randomized controlled trial | Wrong study design |
| 9 | Keshri 2018 | Hypertension and its severity in children with steroid sensitive nephrotic syndrome during remission | Wrong study design |
| 10 | Kniazewska 2009 | Atherosclerosis risk factors in young patients formerly treated for idiopathic nephrotic syndrome | Wrong study design/ Adult population |
| 11 | Leventoglu 2023 | Arterial Stiffness and Cardiac Functions in Children with Idiopathic Nephrotic Syndrome | Wrong study design |
| 12 | Mushina 2023 | Factors Associated with Hypertension and Cardiovascular Parameters in Children with Infrequently Relapsing Nephrotic Syndrome | Wrong study design |
| 13 | Ozdemir 2015 | Bioimpedance for assessing volume status in children with nephrotic syndrome | Wrong study design |
| 14 | Sankar 2016 | Ambulatory Blood Pressure Monitoring in Frequently Relapsing Nephrotic Syndrome | Wrong study design |
