# Supplementary material for: Assessment of subclinical atherosclerosis and cardiovascular disease in children and adolescents with nephrotic syndrome: a systematic review and meta-analysis
Source: Pediatr Nephrol. 2026 Mar 25;41(11):3853–66. doi: 10.1007/s00467-026-07238-1 (PMC13612619; doi:10.1007/s00467-026-07238-1)
Supplement: Supplementary file 3 [file 467_2026_7238_MOESM22_ESM.docx]

| Study | Population | Matching Criteria | Exclusion criteria/Notes |
| --- | --- | --- | --- |
| Ahmed et al. | Healthy children from general outpatient clinic | Age & Sex | Family history of early atherosclerosis  CKD  cardiac anomalies  severe edema  lipid-lowering drugs |
| Alves et al. | Healthy children/adolescents from outpatient clinic | Age & Sex | obesity/underweight, acute/chronic diseases, use of bronchodilators or vasoactive drugs |
| Büyükavci et al. | Healthy children | Age & Sex | N/A |
| Candan et al. | Healthy volunteers | Age & Sex | N/A |
| Chabey et al. | Healthy children from outpatient clinic | Age & Sex | N/A |
| Das et al. | Healthy children from outpatient clinic | Sex only | BMI < 85th percentile, dyslipidemia |
| Elsehmawy et al. | Healthy children | Age & Sex | Heart disease,  Systemic vasculitis, CKD,  severe edema,  lipid-lowering drugs |
| Hooman et al. | Children with history of urinary tract infection from outpatient clinic | Age & Sex | N/A |
| Kamel et al. | Healthy children from outpatient clinic | Age & Sex | N/A |
| Kari et al. | Healthy children | Age only | N/A |
| Mehta et al. | Healthy children | Age & Sex | N/A |
| Nalcalioglou et al. | Healthy children from outpatient clinic | Age & Sex | Conditions or medications affecting renal/cardiac function |
| Papirovic et al. | Healthy children from outpatient clinic | Age & Sex | Medication use |
| Rahul et al. | Children with minor ailments from outpatient clinic | Age only | **Inclusion Criteria:**  Normal BP, BMI 5th–85th percentile, negative family history of premature CVD, normal urine albumin and lipid profile |
| Skrzypczyk et al. | Healthy children | Age & Sex | N/A |
| Yousef et al. | Healthy children from outpatient clinic | Age & Sex | **Inclusion Criteria:**  Normal kidney function; **Exclusion Criteria:**  family history of premature CVD, elevated creatinine, infections,  inflammatory disorders, statin use |
